# Plasticity and artificial selection for developmental mode in a poecilogonous sea slug

**DOI:** 10.1002/ece3.8136

**Published:** 2021-09-21

**Authors:** Serena A. Caplins

**Affiliations:** ^1^ University of California Davis Davis CA USA

**Keywords:** developmental mode, lecithotrophy, planktotrophy, plasticity, quantitative genetics, selection

## Abstract

The contribution of phenotypically plastic traits to evolution depends on the degree of environmental influence on the target of selection (the phenotype) as well as the underlying genetic structure of the trait and plastic response. Likewise, maternal effects can help or hinder evolution through affects to the response to selection. The sacoglossan sea slug *Alderia willowi* exhibits intraspecific variation for developmental mode (= poecilogony) that is environmentally modulated with populations producing more yolk‐feeding (lecithotrophic) larvae during the summer, and more planktonic‐feeding (planktotrophic) larvae in the winter. I found significant family‐level variation in the reaction norms between 17 maternal families of *A. willowi* when reared in a split‐brood design in low (16 ppt) versus high (32 ppt) salinity, conditions which mimic seasonal variation in salinity of natural populations. I documented a significant response to selection for lecithotrophic larvae in high and low salinity. The slope of the reaction norm was maintained following one generation of selection for lecithotrophy. When the maternal environment was controlled in the laboratory, I found significant maternal effects, which reduced the response to selection. These results suggest there is standing genetic variation for egg‐mass type in *A. willowi,* but the ability of selection to act on that variation may depend on the environment in which the phenotype is expressed in preceding generations.

## Graphical abstract

The sacoglossan sea slug *Alderia willowi* exhibits variation in egg size leading to two developmental modes, lecithotrophy and planktotrophy, with differing developmental durations and dispersal potentials. This study confirms experimentally that variation in the production of one offspring type over another is conditional on ambient salinity, with the response varying across families indicating a strong genotype by environment interaction, which can respond to selection, and is influenced by maternal effects. The results reported in this paper suggest the flexibility in developmental mode seen in *A. willowi* is due to an interplay between phenotypic plasticity, directional selection, and maternal effects.

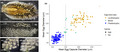

## 1 INTRODUCTION

The evolutionary role of plasticity is highly context‐dependent, sometimes fueling evolution by moving the mean phenotype in the direction favored by selection and other times hindering evolution through the lack of a genetic response to selection on a variable phenotype (see Pfennig et al., 2010). Within a generation, phenotypic variation can shift after a selective event (e.g., a sudden change in environment, or predation), but the response to selection in the following generation reveals whether there is sufficient genetic variation underlying phenotypic variation for evolution to occur (Falconer, 1960; Schlichting & Pigliucci, 1998). Selection experiments can thus reveal the extent to which there is heritable genetic variation for a plastic phenotype and are a powerful means of exploring the potential for adaptive evolution under highly controlled environmental conditions, simplifying the study of environmentally influenced quantitative traits (Fuller et al., 2005; Scheiner, 2002). Selection experiments can also reveal the effect of specific environmental factors, which may influence the response to selection by either revealing or masking “cryptic” genetic variants (Falconer, 1960; Paaby & Rockman, 2014) or by revealing the impact of maternal effects on the response to selection (Kuijper & Hoyle, 2015; McAdam & Boutin, 2004).

Plasticity can have transgenerational effects as the maternal environment can also play a role on the offspring phenotype through maternal effects. Maternal effects have also been shown to facilitate or hinder adaptative evolution depending largely on the predictability of environmental variation (Burgess & Marshall, 2011; Donohue & Schmitt, 1998; Galloway & Etterson, 2007). How maternal effects impact the response to selection has been explored experimentally (Galloway, 1995; Galloway & Burgess, 2009) and theoretically (Kirkpatrick & Lande, 1989; McGlothlin & Brodie, 2009; McGlothlin & Galloway, 2013). Incorporating maternal effects into quantitative genetic models results in stronger evolutionary predictions (Kirkpatrick & Lande, 1989), particularly when tested experimentally (McGlothlin & Galloway, 2013).

Marine invertebrates exhibit astonishing levels of morphological diversity in their adult forms. Their larvae, however, can be broadly grouped into a few developmental modes that, while also morphologically variable, share many functional similarities within and between phyla (Collin & Moran, 2017; Strathmann, 1978; Thorson, 1950). The inferred ancestral state for many phyla is planktotrophic development, involving the production of many relatively small larvae that feed on plankton for weeks to months prior to settlement and metamorphosis to the adult form (McHugh & Rouse, 1998; Strathmann, 1978). Lecithotrophy (nonfeeding) is the most common alternative to planktotrophic development, in which a few relatively large larvae contain substantial amounts of yolk, such that they do not need to feed on plankton before metamorphosis (Marshall et al., 2017; McEdward & Janies, 1997). These two modes can have drastically different influences on larval dispersal and thus may impact micro‐ and macro‐evolutionary patterns and processes, including gene flow, local adaptation, and speciation and extinction (Ellingson & Krug, 2016; Fobert et al., 2019; Grosberg & Cunningham, 2001; Krug et al., 2015). Species that are polymorphic for the type of larvae they produce provide a novel means of addressing the evolution of macro‐evolutionary patterns in a micro‐evolutionary framework. This polymorphism, termed *poecilogony*, occurs when a single species produces both planktotrophic and lecithotrophic larvae (Knott & McHugh, 2012). Intermediates between planktotrophy and lecithotrophy are rare and include lecithotrophic larvae that facultatively feed on plankton (Armstrong & Grosberg, 2018; Armstrong & Lessios, 2015) as well as poecilogonous species and are unlikely to be evolutionarily stable strategies (Knott & McHugh, 2012). In most poecilogonous species, poecilogony is a fixed dimorphism with individuals producing one type of larvae over their lifespan (lecithotrophic or planktotrophic; Levin et al., 1991). In the pocilogonous polychaete annelid *Streblospio benedicti,* forward genetic crosses have shown that larval feeding mode and egg size are able to evolve independently, as these traits occupy different linkage groups (Zakas et al., 2018; Zakas & Rockman, 2014). It has been suggested that in the evolution of developmental mode, egg size is likely one of the first characters to change and is a necessary prior to the reduction or loss of feeding structures or change in feeding behavior seen in some lecithotrophic larvae (Jeffery et al., 2003; McEdward & Janies, 1997; Zakas et al., 2018).

Egg size is a maternally determined quantitative trait (Jha et al., 2015; Moran & Mcalister, 2009) that can be phenotypically plastic and thus influenced by the environment experienced by the egg layer (Collin, 2012a; Fischer et al., 2003; Giménez & Anger, 2001). The extent to which egg size plasticity influences developmental mode evolution is unclear. In marine, invertebrates’ salinity, temperature, and nutrient availability influence egg size within species (e.g., crustaceans, Giménez & Anger, 2001; gastropods, Collin, 2012a), but does not have a documented effect on developmental mode (i.e., whether a larvae needs to feed or not). There is only one poecilogonous species that exhibits environmental modulation in its expression of developmental mode, the sea slug *Alderia willowi*. In *A. willowi*, egg size and number are negatively correlated and bimodally distributed, with individual clutches consisting of either many small eggs that develop into planktotrophic larvae, or relatively few large eggs that while also capable of feeding, can successfully metamorphose into juvenile slugs without feeding on plankton and are thus lecithotrophic in development (Krug, 1998, 2001). The relative frequency of clutches containing either planktotrophically or lecithotrophically developing eggs varies seasonally with more lecithotrophic egg masses produced in the summer months (June–September; Ellingson & Krug, 2016; Krug et al., 2012). In estuarine environments along the California coast, temperature, salinity, and photoperiod all vary seasonally and in laboratory experiments temperature and salinity influence egg‐mass type in *A. willowi* (Krug et al., 2012).

In this paper, I assess the role of plasticity on the evolution of egg‐mass type in populations of the sea slug *A. willowi*. Salinity and temperature both influence the type of egg mass produced by adults and in nature vary predictably with egg‐mass type across seasons (Krug et al., 2012). However, for the experiments presented in this paper, I chose to focus on the effects of salinity. Variation in salinity often presents challenging conditions for estuarine organisms (Giménez & Anger, 2001, 2003). Low salinity can slow larval development or reduce larval survival in many intertidal organisms (Chaparro et al., 2014; Przeslawski, 2004; Sanford et al., 2006) showing that salinity is likely an important selective pressure for intertidal and estuarine organisms. Here, I examine the extent of genetic variation and environmental influence on developmental mode in 17 maternal families of *A. willowi* reared in low and high salinity that reflect mean winter and summer salinities, respectively. I measure the response to selection for lecithotrophy in a quasinatural (Scheiner, 2002) selection experiment within the two salinity environments and evaluate whether one generation of selection affects the direction or degree of plasticity. With these experiments, I seek to describe the relationship between genetics and the environment on egg‐mass type and determine whether there is standing genetic variation through a response to selection for egg‐mass type in a species that exhibits unmatched flexibility for developmental mode.

## 2 METHODS

### 2.1 Study overview

**FIGURE 1:**
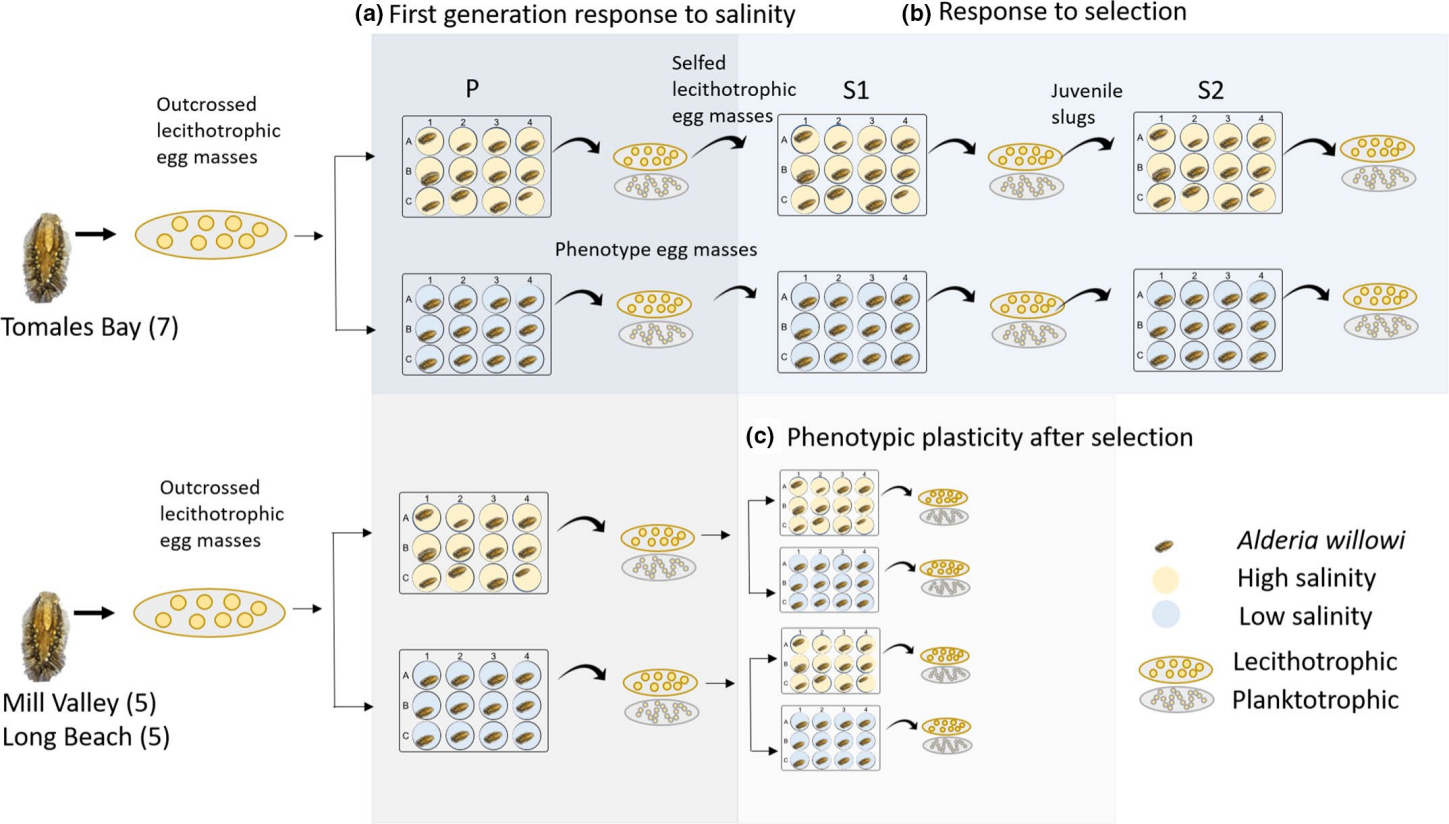
Schematic showing the layout of two split‐brood experiments the data for which addresses three main points. The first‐generation response to salinity (a) uses pooled data from both experiments. The response to selection (b) was estimated from the first experiment using 7 maternal families collected from Tomales Bay. In the second experiment, I evaluate the effect to plasticity following one generation of selection (c) and self‐fertilization in low and high salinity using maternal families from Mill Valley and Long Beach

This paper presents data from two experiments, one designed to evaluate the response to selection in high and low salinity, and the other to determine the role of maternal effects and effect of selection on phenotypic plasticity. The starting conditions for both of these experiments were identical in that they involved offspring from out‐crossed egg masses from field‐collected adults that were reared in laboratory conditions. The results from the first part of these two experiments were pooled and presented as the “first generation response to salinity” (Figure 1). The first experiment explores the response to selection for lecithotrophy in low and high salinity for three generations. Slugs for the first experiment were all collected from a single population in Tomales Bay (northern California). The second experiment uses slugs from two sites, a northern site (Mill Valley) and a southern site (Long Beach) to determine the influence of maternal effects and the effect of one generation of selection on phenotypic plasticity. Finally, I use pooled data from both experiments to explore trait lability, specifically whether individual slugs change the type of egg mass laid.

### 2.2 Study system

**FIGURE 2:**
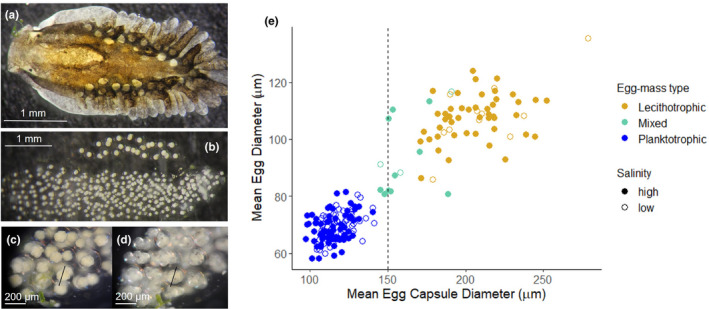
Images showing the study organism, the sacoglossan sea slug *Alderia willowi* (a). The two types of egg masses produced by this species with a lecithotrophic egg mass above a planktotrophic egg mass (b). Egg capsule size is constant through development. Panels C and D show the same embryo at the 32–64 cell stage (c) and 4 days later at the veliger stage (d). At each time, the egg capsule was measured to be 206 µm. Panel (e) shows that the relationship between mean egg diameter and mean egg capsule diameter for a given egg mass is positive (*r* ^2^ = 85, *p*‐value < 2.2e‐16). Salinity does not change the range in egg diameter or egg capsule size but does shift the proportion of egg‐mass type. All images were taken by the author

The sacoglossan sea slug *A. willowi* (Figure 2a) can be found in the upper intertidal zone in estuarian mudflats from Bodega Bay to Sand Diego. These habitats are subjected to seasonal influxes of fresh water during the winter rainy season, with northern sites receiving on average significantly more rain than southern sites (Koch, 2012). Specifically, average yearly precipitation (2007–2019) in Long Beach is 31.14 cm, while in San Francisco, the northern range limit of *A. willowi* is 60.1 cm (NOAA climatic data). Slug populations exhibit local adaptation with northern populations able to survive longer in critically low salinity than southern populations (2 ppt, Koch, 2012), suggesting that salinity is an important selective factor that may influence larval type independent of temperature which also varies seasonally and geographically.

An *A*. *willowi* egg mass consists of dozens to hundreds of eggs strung together and surrounded by a thick jelly‐like substance (Figure 2b). Each individual egg is surrounded by a transparent capsule the diameter of which scales closely with egg diameter (Figure 2e). In *A. willowi*, egg size is correlated with developmental mode, and large eggs (mean ± *SD*: 105 ± 5 µm) develop into lecithotrophic larvae that metamorphose into juvenile slugs in ~5 days, whereas small eggs (mean ± *SD*: 68 ± 4 µm) give rise to planktotrophic larvae that only become metamorphically competent after 30 days of feeding on planktonic algae (Krug, 1998). Slugs spend approximately 2–3 weeks as juveniles before they lay their first egg mass (Smolensky et al., 2009). Both size classes of larvae can feed on phytoplankton, but the larger, lecithotrophically developing larvae do not need to feed to complete metamorphosis and occasionally develop into the juvenile stage while still encapsulated in their egg capsule, bypassing a swimming stage altogether (Botello & Krug, 2006; Krug, 2001). Infrequently, field‐collected individual *A. willowi* produce mixed‐egg clutches containing both lecithotrophic and planktotrophic embryos (Krug, 1998). In these egg masses, larvae with a larval shell diameter >160 µm exhibit lecithotrophic development, whereas smaller larvae are all planktotrophic (Krug, 1998). The type of egg mass laid is influenced by the rearing conditions experienced by juvenile slugs as they develop into adults and thus acts as a seasonal polyphenism through maternal effects (Krug et al., 2012). The lability of egg mass type in *A. willowi* (i.e., individual plasticity) has been reported in response to laboratory conditions and starvation for slugs freshly collected from the field, and in these cases is always asymmetrical (lecithotrophic laying slugs transition to laying planktotrophic egg masses, Krug, 1998). For slugs reared entirely in the laboratory, “switching” has also been reported when laboratory conditions were attempted to remain constant (Smolensky et al., 2009). Thus, it appears egg‐mass type is labile but the environmental conditions under which slugs change egg‐mass type and the effect this may have in natural populations is unclear. To monitor individual variation in egg‐mass type, I performed all of the experiments in this paper with slugs reared individually.

Populations of *A. willowi* are found on mudflats in estuarine environments and can be extremely variable in density, from several dozen individuals/m^2^ to 1,300 slugs/m^2^ (Garchow, 2010). Individuals are typically polyandrous, with multiple matings via hypodermic insemination (Smolensky et al., 2009). At low densities, however, *A. willowi* exhibits “delayed selfing” (Smolensky et al., 2009). Self‐fertilized egg masses are occasionally incompletely fertilized and *A. willowi* will continually deposit unfertilized or partially fertilized egg masses when reared in isolation (personal obvs., Smolensky et al., 2009).

### 2.3 First‐generation response to salinity

Adult *A. willowi* were collected from three sites in CA: Tomales Bay (20 June 2017 [38°06′59″N 122°51′16″W], Mill Valley (12 September 2017 [37°52′55″N 122°31′03″W], and Long Beach (14 September 2017, provided by Patrick Krug [33.73 N, 118.203 W]). I selected lecithotrophic egg masses from 17 adult slugs (Tomales Bay: 7; Mill Valley: 5; Long Beach: 5), from which the embryos constituted 17 maternal families consisting of unknown mixtures of full‐ or half‐sibs. Embryos from each family were hatched and reared to the newly metamorphosed juvenile stage in 32 ppt filtered seawater at room temperature. Embryos were not provided with planktonic algae and instead relied on their yolk stores to complete development. Once slugs were at the crawling juvenile stage, I haphazardly selected 24–36 individuals from each maternal family to either the low (16 ppt) or high (32 ppt) salinity treatment. Slugs were reared individually in 12‐well culture dishes with 5‐ml volume per well and on a 14L:10D light cycle at room temperature (22 ± 2℃). I covered each culture plate with plastic wrap that has a water‐resistant adhesive on one side to keep slugs in their respective wells. Three times weekly, I fed slugs freshly collected algae (*Vaucheria longicaulis*), carried out 50% water changes, and checked for newly deposited egg masses. Once slugs reached maturity and began laying egg masses, I photographed each egg mass using a Nikon CoolPix P7100 on a Wild Heerbrugg dissecting microscope at 50× magnification. I measured the diameter of three egg capsules for egg masses that contained eggs of just one size class and measured six egg capsules (three of each size class) for egg masses that contained both small and large eggs in ImageJ (v1.52). To show the relationship between egg‐capsule diameter and egg diameter, for every egg mass that contained eggs that had yet to cleave, I measured the diameter of three to six eggs (as above) in addition to measuring the egg‐capsule diameter. To test the association between egg diameter and egg‐capsule size, I ran a linear model with egg diameter as the predictor and egg‐capsule size the response.

As egg size can only be accurately measured prior to embryonic cleavage, and thus within the first 1–2 hr postoviposition, for most of the data presented in this paper, I used egg‐capsule size as a proxy for developmental mode. I categorized developmental mode according to egg capsule size in a clutch/egg mass, assuming that egg capsules ≥150 µm develop lecithotrophically as experimentally confirmed by Krug (1998). I used an R script to confirm which egg masses were “mixed” based on egg capsule measurements. I verified these “mixed” egg masses through examination of the egg‐mass images.

### 2.4 Selection for lecithotrophy in low and high salinity

To evaluate the response to selection for lecithotrophy, I selected egg masses containing large eggs for three generations using maternal lines that were collected from Tomales Bay, CA (Figure 1b). The larvae from selected egg masses were at no time fed planktonic algae, and thus, all that survived to the juvenile stage were lecithotrophic in their development in that they came from large eggs and did not need to feed as larvae. The S_1_ and S_2_ generations were the product of self‐fertilization, because the hermaphroditic slugs were raised in isolation and thus are denoted with an “S” instead of the traditional “F” for cross‐fertilized offspring. Control lineages, lineages where selection for larval type is not applied, were not included due to the experimental intractability of the difference in degree of care and generation time of the two larval types. Slugs were reared in 12‐well cell culture plates, which were covered with plastic wrap as described above. I fed adult and juvenile slugs *V. longicaulis* and changed their water three times weekly. I measured egg capsule size for five capsules per egg mass in ImageJ (v1.52a). I calculated realized heritability on egg capsule size as well as on the proportion of lecithotrophy using the breeder's equation (*R* = *h* ^2^ *S*). For the proportion of lecithotrophy, I modified the breeder's equation for a threshold response using a probit transformation to translate the proportion of individuals expressing the trait of interest to a mean value for that trait (Walsh & Lynch, 2018).

### 2.5 Selection, the reaction norm, and maternal effects

To evaluate whether the slope of the reaction norm changes following selection for lecithotrophy, I reared slugs from the S_1_ generation from Mill Valley and Long Beach in either low or high salinity. Mill Valley and Long Beach are the northern and central range sites for *A. willowi*, respectively, and may be different in their response to salinity due to differences in seasonal annual rainfall (Garchow, 2010). Fifty percent of every clutch was reared in either high or low salinity, as described previously. I measured egg capsule size for three to six egg capsules per egg mass in ImageJ (v1.52a). I tested the significance of the parental environment (i.e., maternal effects) on the response to selection using a linear model with the response egg capsule diameter against the predictors salinity and generation (pre‐ or postselection, see Ezard et al., 2014; Kirkpatrik & Lande, 1989).

### 2.6 Analysis of genetic variance and heritability

Models of quantitative genetics use population pedigree information to estimate genetic variance and heritability. Standard models of quantitative genetics assume traits have normal distributions; however, many traits are non‐normally distributed (Hadfield, 2010). Generalized linear mixed models (GLMM) make use of a latent variable (ℓ) rather than the observed response, and in simulated data provide a better fit for binary traits than parent–offspring regression (de Villemereuil, 2012). The latent variable of GLMMs incorporates non‐normal trait distributions in quantitative genetics models. In this paper, I model salinity as a fixed effect and individual (ID), family (maternal effect), and 12‐well dish, as random effects with collection site and family as nested random effects (i.e., random = ~ID + Family + dish + Family:CollectionSite). By including in the model the effect of collection site as a nested random variable, I account for the fact that site‐specific maternal effects may play a role in egg‐mass type variation via maternal effects (Kawecki and Ebert, 2012). For these models, I used a pedigree that conservatively assumes offspring are maternal half‐sibs as natural populations are typically observed mating in large aggregations (Smolensky et al., 2009). I test the effect of the assumption of within clutch relatedness on genetic variance by running the same models with simulated relationships as half‐sibs, full‐sibs (outcrossed), full‐sibs (selfed), and heterogeneous mixtures of relatedness. I tested the effect of salinity on egg‐mass type with the response either as a continuous variable with a Gaussian distribution for egg‐capsule size or as a binary variable for developmental mode (e.g., lecithotrophy = 1, planktotrophy = 0). For the binary analysis, I calculated the mean egg capsule size per individual slug. I scored mean values >150 µm as lecithotrophic and mean values <150 µm as planktotrophic and was thus analyzed as a threshold trait (i.e., a quantitative trait with discrete expression, see Roff, 1996). I also present the results of treating developmental mode as a categorical trait with three categories: lecithotrophic, planktotrophic, and mixed. Because the categorical traits (developmental mode) are derived from the continuous trait (egg capsule size), I performed each analysis separately and not as a multivariate analysis. A GLMM requires a probit link function to go from the latent Gaussian variable to the observed response variable. In the case of a threshold response, this takes the form: $P\left(y_{i}^{0,1}=1\right)={\text{probit}}^{‐1}\left(\ell_{i}\right)$. The link function for the response of egg capsule size took the standard form for a Gaussian response variable (see de Villemereuil, 2018). The models were run in the R package *MCMCglmm* (Hadfield, 2010). I specified priors for the Gaussian model (egg capsule size) as a normal distribution with mean = zero and a small variance (1), and for the threshold models (binary and categorical) as a normal distribution with mean = zero and a large variance (1,000) with a link variance (V_1_) of 1, as described in de Villemereuil (2018). All analyses were performed in the R environment (v3.5.1, R Core Team, 2013), and the code used along with all the data presented in this paper are available on Dryad (https://doi.org/10.25338/B8JK9Q).

### 2.7 Broad‐sense heritability

Heritability for threshold traits can be measured on two scales, the observed non‐normally distributed phenotypic scale, and the normally distributed unobserved *liability* (Falconer, 1960; de Villemereuil, 2018). I used the R package *QGglmm* (de Villemereuil, 2018) to calculate heritability on both the observed and liability scale for developmental mode and on just the observed scale for egg‐capsule diameter. I analyzed developmental mode as a binary trait (lecithotrophy = 1, planktotrophic = 0) and as a categorical trait (lecithotrophic, planktotrophic, and mixed) in a MCMCglmm model set for a “threshold” distribution (or “categorical,” respectively) for 603,000 iterations with a burn‐in phase of 10,000 and a thinning interval of 10.

### 2.8 Genetic correlation

Falconer (1960) noted that a phenotype produced in two environments could be viewed as two separate phenotypes, and thus, a genetic correlation can be calculated between the two. This correlation can be used to determine the degree to which a phenotypic response is influenced by the environment, where a perfect correlation (= 1) between environments indicates zero environmental influence. This correlation also provides a prediction for how a given phenotype may respond to selection in a given environment (Falconer, 1952). I used the family‐level proportion of lecithotrophic egg masses produced in low and high salinity to evaluate the genetic correlation between salinities (see Roff, 1996; Via, 1984).

## 3 RESULTS

### 3.1 First‐generation response to salinity

**FIGURE 3:**
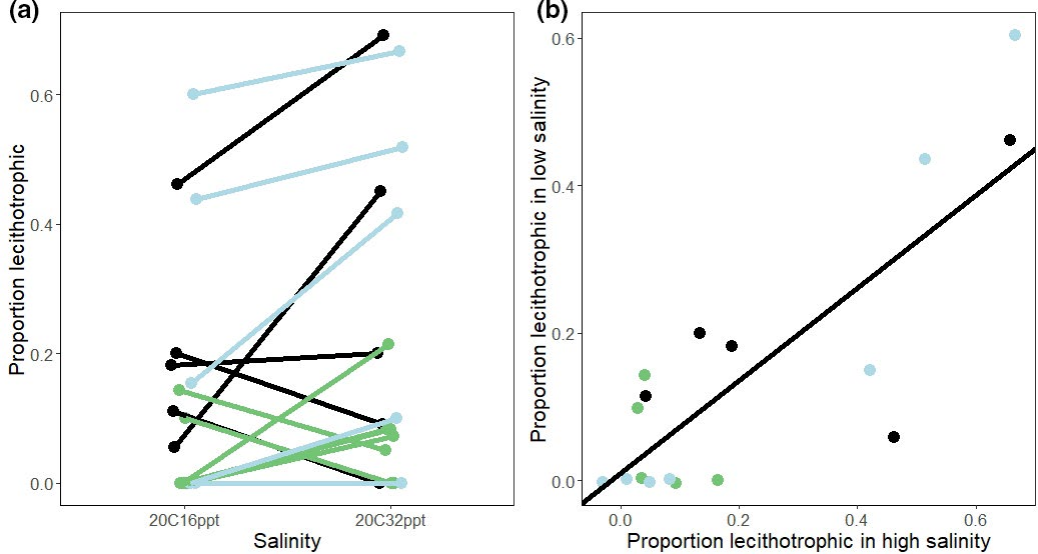
(a) Family reaction norm for the proportion of lecithotrophy in low (16 ppt) and high (32 ppt) salinity and (b) the correlation between families for the proportion of lecithotrophy produced in low and high salinity (slope = 0.63, intercept = 0.001). Each line in a and each dot in b are a maternal family. Colors denote sampling sites: Tomales Bay, CA in green, Mill Valley, CA in light blue, and Long Beach, CA in black. Points and lines were jittered slightly to show all the points, as some families laid the same proportion of lecithotrophic egg masses

Egg capsule size closely predicts egg size (lm, *r* ^2^ .85, *p*‐value < 2.2e‐16), and egg size is a proxy for developmental mode (Figure 2e; Krug, 1998). Egg capsule size and egg size can be measured on a continuous scale, but both are bimodally distributed (Figures 2e and 3). Egg size has a smaller standard deviation than egg capsule size measured across all egg masses classes (Table 1) Egg capsule size remains constant throughout development (Figure 2c,d). Slugs began to deposit egg masses when they were an average of 17.5 days old. A total of 2,846 egg masses were laid by 433 slugs from 17 families. On average, in the first generation, each family consisted of 26 individuals (median: 25, *SD*: 8.7, range: 10–43). The number of egg masses an individual laid ranged widely (mean = 4.5, min = 0, max = 33). Most egg capsules within an egg mass were similar in size (mean = 136 µm, *SD* = 9.7, and the two highest modes = 113, and 182 µm). There was not a significant difference in the range of egg‐capsule sizes for whether they were laid in high or low salinity (Table 1; Figure 2e).

**TABLE 1:** First‐generation response to salinity, showing the egg diameter, egg‐capsule diameter, the proportion of lecithotrophy, and mixed egg masses and the total number of egg masses laid in each treatment

Trait	Low salinity	High salinity
Egg diameter mean (± *SD*)	77 (± 16)	85 (± 19)
Egg‐capsule diameter mean (± *SD*)	128 (± 33)	137 (± 41)
Egg‐capsule size range	66–305	68–359
% Lecithotrophic egg masses	17.60%	25.70%
% Mixed egg masses	6.30%	6.70%
Number laid	1,037	1,809

Note

Data for this table were pooled from two experiments and from slugs collected from three sites (see Figure 1).

The reaction norm of the proportion of lecithotrophic egg masses reveals considerable variation for egg‐mass type within and between families (Figure 3a). Most families show an increase in egg capsule size and in the proportion of lecithotrophic eggs in high salinity, although four families produced more lecithotrophic egg masses in low salinity than in high (Figure 3a, showing proportion lecithotrophic). Five families produced lecithotrophic egg masses in high salinity, but not in low salinity (Figure 3a,b). Likewise, one family produced no lecithotrophic eggs in either salinity, but also had the lowest survival rate in laboratory conditions of any other family. Three of the four reaction norms with negative slopes had a small sample size due to low survival in experimental conditions (*N* < 10). Offspring survival to adulthood was lower in low salinity than in high salinity (63% vs. 81%, respectively). While survival declined in low salinity, survival was not significantly correlated with the proportion of lecithotrophy in either low or high salinity (linear model, low salinity *r* ^2^ = .001, *p* = .89; high salinity r^2^ = .005, *p* = .77).

### 3.2 Genetic correlations between environments

The family response to salinity is positively correlated across salinity treatments (Figure 3b; slope = 0.63; Y‐intercept = 0.001, multiple *r* ^2^ = .89, *p*‐value 7.13e‐05). This slope predicts the expected response to selection for developmental mode between high and low salinity: for every one‐unit change in response to selection in high salinity, a corresponding 63% change should occur in low salinity.

### 3.3 Analysis of genetic variance and heritability

The analysis for egg capsule size revealed a significant effect of salinity (MCMCglmm for Gaussian trait; salinity *p*‐value = .006). Broad‐sense heritability for egg capsule size was 0.532 (Table 2). I did not find an effect of including the random effect of maternal family nested within collection site (DIC with nested effect = −25,289.2, DIC without = −25,289.6). The model testing the fixed effect of salinity, and the random nested effect of maternal family and region on the proportion of lecithotrophic egg masses also revealed a significant effect of salinity (MCMCglmm for a threshold trait, salinity *p*‐value < 2e‐04). Broad‐sense heritability for the proportion of lecithotrophy on the observed scale was 0.229 and on the latent scale was 0.453 (Table 2). Finally, the categorical model which includes three egg‐mass types (lecithotrophic, planktotrophic, and mixed) found again a significant effect of salinity (MCMCglmm for categorical trait, *p*‐value = 8e‐05). Using the categorical model, I calculated heritability for the proportion of lecithotrophic, planktotrophic, or mixed egg masses as 0.33, 0.39, and 0.05, respectively (Table 2). For the first two models, I assessed model fit by confirming that the effective sample size exceeded 1,000, and the trace and density plots showed adequate mixing. The categorical model did not obtain an effective sample size greater than 1,000 (= 350), but the trace and density plots were well mixed.

**TABLE 2:** Summary of model values and broad‐sense heritability where *V_a_* is the additive genetic variance, *V_r_* is the latent residual link variance as computed in each model, *µ* is the latent intercept, *H* ^2^ latent is the ratio of *V_a_* over the sum of *V_a_* and *V_r_* plus the variance of a normal distribution (= 1), and *H* ^2^ observed is calculated from the observed data in QGglmm (see de Villemereuil, 2018)

Trait	Distribution	*µ*	*V_a_*	*V_r_*	*H* ^2^ latent	*H* ^2^ obs.
Egg‐capsule size	Gaussian	0.13	0.00086	0.00073	NA	0.538
Egg‐mass type	Binary	0.206	0.163	1	0.454	0.223
Egg‐mass type	Categorical	3.9	12.7	13.7	0.93	L = 0.33 *p* = .39 *M* = 0.05

Note

These values were calculated assuming egg clutches were maternal half‐sibs using the pooled first‐generation response to salinity data from all three collection sites (Tomales Bay, Mill Valley, and Long Beach). Egg‐mass type was analyzed as a binary (lecithotrophic = 1, planktotrophic = 0) and categorical trait (L = lecithotrophic, P = planktotrophic, *M* = mixed) to account for different egg‐mass types, where the categorical model provides an estimate of heritability for each egg‐mass type.

### 3.4 Selection for lecithotrophy in low and high salinity

**FIGURE 4:**
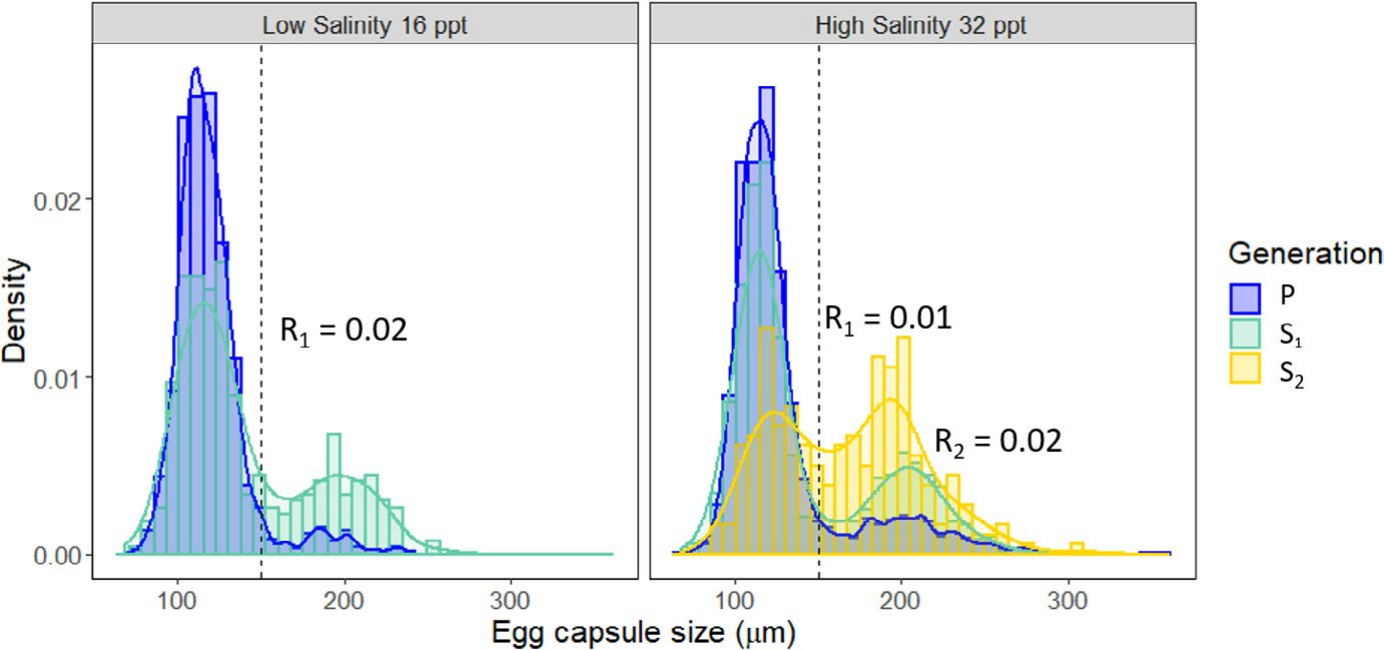
Barplot with density overlay showing the response to selection for lecithotrophy across several generations in low (16 ppt) and high (32 ppt) salinity from slugs collected from Tomales Bay (Figure 1b). The vertical dashed line indicates the cutoff for lecithotrophic or planktotrophic development (egg capsule size >150 µm). Generations S_1_ and S_2_ are “selfed” (see Methods), while the parental generation is the product of outcrossing in the field. The S_2_ generation in low salinity is not shown due to small sample size (4 individuals from a single family, all of which laid lecithotrophic egg masses). The response to selection is shown for each generation on the plot where R_1_ refers to the response from parental to S_1_, and R_2_ is the response from S_1_ to S_2_

Selection for lecithotrophic egg masses across three generations resulted in a proportional increase in the number of lecithotrophic egg masses in both low and high salinity (Figure 4; Table 3). As the sample size for the low‐salinity S_2_ generation was very small (1 family line, 4 individuals) only the S_2_ generation for the high salinity treatment is shown (three family lines, 17 individuals). The small sample size of the low salinity S_1_ and S_2_ generations is compounded by low salinity slugs having a lower survival rate and producing a smaller fraction of self‐fertilized lecithotrophic egg masses. The response to selection was similar for both low‐ and high‐salinity selected lines, while the selection coefficient was greater for the low‐salinity selected lines (Table 4). Selection increased the proportion of mixed egg masses in both low and high salinities (Table 3; Figure 4). The summed realized heritability for egg capsule size was 0.39 for high salinity and 0.34 for low salinity (Table 3). Similarly, for developmental mode, realized heritability was 0.35 for high salinity and 0.38 for low salinity (Tables 3 and 4).

**TABLE 3:** The response to selection for lecithotrophy in low (16 ppt) and high (32 ppt) salinity for slugs collected from Tomales Bay (TB)

Generation	Low salinity				High salinity			
	*N* (family)	Mean (± *SD*)	% L	% M	*N* (family)	Mesan (± *SD*)	% L	% M
P (TB)	75 (7)	120 (22)	5%	4.4%	79 (7)	130 (37)	14%	6%
S_1_	34 (2)	14 0(40)	19%	11%	68 (7)	150 (45)	18%	6%
S_2_	NA	NA	NA	NA	17 (3)	170 (43)	42%	18%

Note

Showing the mean egg‐capsule size (µm) and the proportion of lecithotrophic egg masses (% L) and proportion mixed egg masses (% M). *N* is the number of individuals that survived to lay eggs, with the number of maternal family lines parenthetical to the number of individuals. Realized heritability was calculated for both the trait egg‐capsule size, and the proportion of lecithotrophy and is presented here as the ratio of the sum of the phenotypic and additive variances across generations.

**TABLE 4:** Parameters for realized heritability in low and high salinity for egg capsule size and the proportion of lecithotrophy across three generations of selection

Proportion lecithotrophic	Low salinity					High salinity				
Generation	*q*	*u*	*s*	*R*	*H* ^2^	*q*	*u*	*S*	*R*	*H* ^2^
P	0.10	−1.28	1.75	0.70	0.40	0.19	−0.88	1.43	0.41	0.29
S1	0.28	−0.55				0.32	−0.47	1.12	0.49	0.44
S2						0.51	0.03			
Sum *H* ^2^										0.35
Egg capsule size										
Generation		*u* (µm)	*S*	*R*	*H* ^2^		*u* (µm)	*S*	*R*	*H* ^2^
P		124	0.06	0.02	0.34		133	0.05	0.01	0.24
S1		144					146	0.03	0.02	0.63
S2							167			
Sum H^2^										0.39

Note

Maternal lines were collected from Tomales Bay. Where *q* is the proportion of lecithotrophy preselection when applicable, *µ* is the mean trait value, *S* is the selection coefficient, *R* is the response, and *H* ^2^ is the broad‐sense heritability. Sum *H* ^2^ is calculated by dividing the summed responses by the summed selection coefficients for each trait and salinity separately.

**FIGURE 5:**
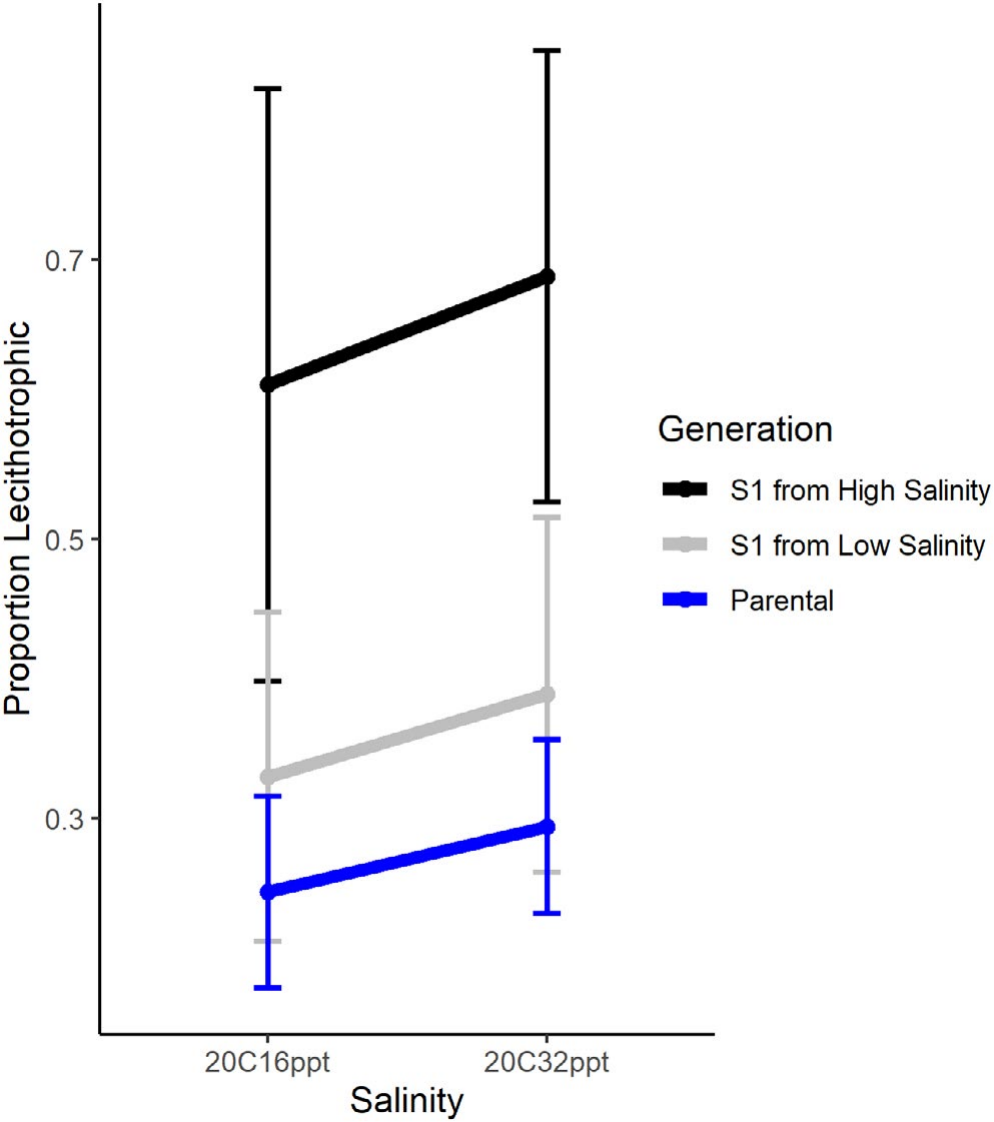
The reaction norm before and after one generation of selection for lecithotrophy in low and high salinity, from slugs collected from Long Beach and Mill Valley. The proportion lecithotrophic is the mean proportion between families and the error bars show standard deviation. The response to selection in high salinity was significant, but was not significant for low salinity, indicating a significant effect of the maternal environment (maternal effects)

In the second experiment, maternal effects and the slope of the reaction norm following selection was tested with 349 individuals from 9 families (Long Beach: *n* = 5, Mill Valley: *n* = 4) selected in both low and high salinity (Figure 1c). The slope of the reaction norm remained positive following selection but the degree of change varied significantly with the response to selection greater in high salinity than low (Figure 5, Table 5). There was a significant maternal effect of parental salinity on the response to selection (model: lm (proportion_lecithotrophy ~ generation * rearing_environment − 1, high selection *p*‐value = .000109, low selection *p*‐value = .08814, parental generation (maternal effects) *p*‐value = .03707, S_1_ rearing environment *p*‐value = .88, model *p*‐value = 1.482e‐07, multiple *R* ^2^ = .77, *F* = 15.25).

**TABLE 5:** Egg‐capsule size and the proportion of lecithotrophy for the experiment on the effect of selection on the reaction norm (Figure 4)

Generation	Low salinity				High salinity			
	*N* (family)	Mean (± *SD*)	% L	% M	*N* (family)	Mean (± *SD*)	% L	% M
P (LB, MV)	114 (9)	130 (34)	17%	6%	165 (9)	137 (42)	25%	7%
S_1_ low selected	26 (4)	156 (42)	21%	12%	17 (4)	157 (46)	28%	9%
S_1_ high selected	18 (3)	156 (36)	39%	13%	29 (4)	169 (38)	65%	5.6%

Note

The parental generation (P) was from outcrossed egg masses collected from individuals from Long Beach (LB) and Mill Valley (MV). The number of individuals that survived to lay eggs (*N*) and the number of maternal family lines are parenthetical to the number of individuals. Proportion lecithotrophy is indicated by %L and the proportion of mixed egg masses by %M.

### 3.5 Trait lability

Slugs in both experiments were reared in individual wells to monitor whether they switch the type of egg mass they produced. Out of data from all slugs presented in this study including those from the selection lineages (a total of 622 individuals), most (68%) laid the same type of egg mass throughout the experiment (10% laid lecithotrophic egg masses, 57% planktotrophic and 1% laid mixed egg masses). Of the slugs that switched their egg‐mass type, 12% laid both planktotrophic and lecithotrophic, while 8% laid planktotrophic and mixed egg masses and 3% laid both lecithotrophic and mixed. An additional 8% laid all three egg‐mass types. I found a significant increase in switching in high salinity in the parental generation and a significant interaction between high salinity and the parental generation, but no effect of collection site (glm with “switching” as a binary variable AIC 672.33, high‐salinity *p*‐value = .039, low‐salinity *p*‐value = .33, parental generation *p*‐value < .001, generation S_1_ *p*‐value = .99, generation S_2_ *p*‐value = .98, high salinity: parental generation *p*‐value = .0053, collection site *p*‐value = .46).

## 4 DISCUSSION

The sacoglossan sea slug *A. willowi* exhibits variation in egg size leading to two developmental modes, lecithotrophy and planktotrophy, with differing developmental durations and dispersal potentials. Previous studies have shown that in *A. willowi* intraspecific variation in developmental mode (poecilogony) is a seasonal polyphenism modulated by the environment experienced by juvenile slugs (Krug et al., 2012). This study confirms experimentally that variation in the production of planktotrophic versus lecithotrophic offspring is at least partly conditional on ambient salinity but that the response varies across families, indicating a strong genotype by environment interaction. Egg‐mass type in *A. willowi* responds readily to selection for increased proportions of lecithotrophy implying there is standing genetic variation for developmental mode. Low‐salinity treatments resulted on average in a lower proportion of lecithotrophic egg masses. I present evidence that the response to selection may be influenced by maternal effects (Figure 5). While these results are preliminary due to small sample size and low number of generations, they suggest that the striking flexibility for developmental mode seen in *A. willowi* is due to an interplay between phenotypic plasticity, directional selection, and maternal effects.

Is phenotypic plasticity for egg‐mass type adaptive in *A. willowi*? Egg‐mass type plasticity only occurs once in the *Alderia* genus, for which planktotrophy is the ancestral state and the only other mode of development (i.e., there are no lecithotrophic *Alderia* species, Krug et al., 2015). In *A. willowi*, it appears plasticity for egg‐mass type has evolved alongside adaptations to the higher ambient temperature and less frequent low‐salinity events of their Central and Southern California habitats (Krug et al., 2012, 2021). Salinity is a common stressor for estuarine animals that varies seasonally in California (Cloern et al., 2017). As osmoconformers, low salinity presents metabolically expensive conditions for marine invertebrates (Rivera‐Ingraham & Lignot, 2017). Low‐salinity stress has been identified as the leading factor determining the northern range limit of *A. willowi* with northern populations showing local adaption to more frequent low‐salinity pulses than populations at the range center (Koch, 2012). In this paper, I documented reduced survival in low salinity (possibly via environmentally induced inbreeding depression of self‐fertilized egg masses, see Cheptou & Donohue, 2011). Low salinity leads to significantly longer developmental times and significantly reduced hatching success of both lecithotrophic and planktotrophic *A. willowi* larvae (Krug et al., 2021). Planktotrophic larvae typically hatch from the encapsulated egg mass earlier than lecithotrophic larvae (Krug, 1998) and even under low‐salinity stress (12 ppt) planktotrophic *A. willowi* larvae hatch earlier than lecithotrophic larvae (Krug et al., 2021), possibly reducing their time spent in stressful low‐salinity near‐shore conditions. These data suggest an adaptive role of phenotypic plasticity for egg‐mass type and developmental mode in *A. willowi*, but they do not rule out a maladaptive response or the possibility that developmental mode is an exaptation evolving alongside osmoregulation (or heat tolerance). For example, producing fewer lecithotrophic larvae may be maladaptive if low salinity occurs with a mismatch in adult or larval food sources (termed “selfish maternal‐effects” in Krug et al., 2012; Marshall Dustin et al., 2008).

The scale of environmental heterogeneity as it relates to generation time provides insight into the selective factors that may be driving developmental mode variation in *A. willowi*. Plasticity evolves under conditions that are predictable (Leung et al., 2020). Seasonally varying conditions like temperature, salinity, and photoperiod make effective cues for which plasticity and specifically polyphenism can evolve and be maintained through frequency‐dependent selection (Chevin & Lande, 2013), though climate change is altering many of these patterns (Berg & Hall, 2015). For *A. willowi*, salinity and temperature have been identified as cues that when experienced by juvenile slugs induce a change in the likelihood of the egg‐mass type they will produce as adults (Krug et al., 2012 and this paper). Adult nutrient availability is also a critical factor that influences egg production (Drummond‐Barbosa & Spradling, 2001; Garrido & Barber, 2001). How nutrient availability in nature varies for *A. willowi* requires further study. Adult *A. willowi* obligately consume the alga *Vaucheria longicaulis* which can form large dense patches in the upper intertidal zone of the mudflats. Variation in algal patch abundance appears to be subject to some seasonality and geography (e.g., patches are largest in the summer in northern California, but large patches can occur during the winter months in southern California, Pat Krug, pers. com.), as well as microhabitat effects (e.g., shade, substrate composition (clay, sand, mud)) These seasonal and shorter time scale patterns overlap with generation time in *A. willowi*, which may have up to 12 generations each year (Krug, 1998). Adult lifespan is difficult to determine, but the ephemeral nature of their habitat likely limits the average lifespan to several months (pers obs). Theory supported by several case studies predicts a stronger role of phenotypic plasticity for organisms with long generation times and a greater role of genotypic variation for those with short generations (Bergland et al., 2014). Current estimates of population genetic variation in *A. willowi* are derived from allozymes and single gene sequences (CO1 and 16S) and suggest that there is genetic differentiation between populations (Krug et al., 2007). As plasticity and genetic variation for life‐history traits are not mutually exclusive and I suggest that *A. willowi* is an ideal model system for disentangling these factors.

Correlations between environmental conditions and developmental mode provide a way to formulate testable hypotheses about which variables may act as agents of selection or as cues for inducible phenotypes. Marshall et al. (2012) identified a significant relationship with sea surface temperature and chlorophyl‐a productivity across three categories of developmental mode (planktonic‐feeding, planktonic‐nonfeeding, and aplanktonic). While many invertebrate species adjust egg size in response to abiotic conditions, a subsequent shift in developmental mode has not been reported (i.e., a plastic response that results in feeding larvae no longer requiring food or vice versa) with *A. willowi* being the exception. Regardless, adaptive egg size plasticity in response to temperature is well documented across a wide range of taxa (e.g., *Crepidula* gastropods, Collin, 2012b; *Bicyclus* butterflies, Fischer et al., 2003; and rotifers, Sun & Niu, 2012). There are several examples of salinity‐induced egg size plasticity in estuarine crabs (Collin & Moran, 2017; Giménez & Anger, 2001 and across seasons Collin et al., 2001). Likewise, egg size plasticity was found to be maladaptive under fluctuating thermal environments in seed beetles (Leonard & Lancaster, 2020). These case studies show that the direction and degree of egg size plasticity varies considerably across species and are likely best understood together, as a multivariate response is more reflective of the selective regime under which trait evolution occurred and enhances our ability to determine whether plasticity is adaptive. Likewise, interspecific and intraspecific comparisons of egg size and developmental mode will be enhanced by detailed quantification of egg composition (i.e., protein:lipid ratios, Moran & McAllister, 2009) as we determine the specific agents and targets of selection on developmental mode.

There is robust support for the influence of the early rearing environment on adult egg‐mass type in *A. willowi* (Krug et al., 2012, this paper), However, it is unknown whether the individual trait lability documented in this paper (see also Smolensky et al., 2009) has a genetic or environmental basis and what impact trait lability has for the evolution of developmental mode in *A. willowi*. The contribution of trait lability to developmental mode variation and its potential interactions with family‐level plasticity merit further investigation. The data presented in this paper suggest that there is standing genetic variation for egg‐mass type upon which selection can act, but that the response the selection depends on the environment in which the trait is exhibited and can be greatly influenced by maternal effects. In estimating additive genetic variance, I assumed that clutches contained half‐sibs, which while being a conservative estimate is likely to increase the estimated role of the environment and decrease the estimated role of genetics. This has the potential to increase environmental effects including maternal effects; however, for the calculation using this assumption (the first‐generation response to salinity), I did not find a significant effect of the maternal environment, which was only found in a subsequent experiment and analysis when maternal environment was controlled. Furthermore, in this paper, selection for lecithotrophy takes place alongside self‐fertilization, which could itself increase the proportion of lecithotrophy as a protection against inbreeding depression (Pilakouta et al., 2015) and warrants further study. Finally, the result presented in this paper should be carefully considered in the context of the lack of a control lineage, which is currently precluded by the difficulty in rearing planktotrophic larvae in the laboratory. Thus, the increase in lecithotrophy seen in the laboratory could be due to an adaptation to laboratory conditions apart from selection for increased lecithotrophy.

## 5 CONCLUSION

The diminutive sea slug *A. willowi* exhibits a unique suit of traits, the study of which continues to inform our understanding of phenotypic plasticity, life‐history evolution, seasonal adaptation, dispersal dimorphisms, and maternal effects (Botello & Krug, 2006; Krug et al., 2012, 2015; Smolensky et al., 2009, this paper). In the context of the evolution of lecithotrophy, I present data in this paper that support egg‐mass type as a polygenic and environmentally sensitive trait (i.e., a threshold trait, Roff, 1996). This paper adds to the collection confirming that the juvenile environment plays a significant role in determining where the threshold for egg‐mass type lies and shows the extent to which the maternal environment plays a role in the type of offspring produced the following generation. The complicated interplay between temperature, salinity, and nutrient availability (of both adult and larval food sources) results in highly ephemeral habitats for *A. willowi* and likely act in concert to maintain their flexible life history.

## CONFLICT OF INTEREST

None declared.

## AUTHOR CONTRIBUTION

**Serena A. Caplins:** Conceptualization (lead); Data curation (lead); Formal analysis (lead); Funding acquisition (lead); Investigation (lead); Methodology (lead); Project administration (lead); Resources (lead); Software (lead); Supervision (lead); Validation (lead); Visualization (lead); Writing‐original draft (lead); Writing‐review & editing (lead).

### OPEN RESEARCH BADGES

This article has been awarded Open Data, Open Materials Badges. All materials and data are publicly accessible via the Open Science Framework at https://github.com/SerenaCaplins/GXE_A.willowi. https://doi.org/10.25338/B8JK9Q.
